# Supplementary material for: Highly efficient genome editing by CRISPR-Cpf1 using CRISPR RNA with a uridinylate-rich 3′-overhang
Source: Nat Commun. 2018 Sep 7;9:3651. doi: 10.1038/s41467-018-06129-w (PMC6128929; doi:10.1038/s41467-018-06129-w)
Supplement: Supplementary file 1 — Supplementary Information [file 41467_2018_6129_MOESM1_ESM.pdf]

Supplementary Information

Highly Efficient Genome Editing by CRISPR-Cpf1 using CRISPR RNA with an uridinylate-rich 3'-overhang

Moon & Lee et al.

Contents:

Supplementary Figure 1. Improved AsCpf1 activity by addition of U3 3'-overhang in crRNA.

Supplementary Figure 2. Unbiased in vitro analysis to pursue the optimal crRNA configuration.

Supplementary Figure 3. *Confirmation of the U-rich crRNA as an optimal configuration for AsCpf1 activity.*

Supplementary Figure 4. Improved knock-in efficiency by crRNA carrying the U-rich 3'-overhang.

Supplementary Figure 5. Functionality of Digenome-seq off-target analysis.

Supplementary Figure 6. Uncropped, original images of blots and gels shown in this study.

Supplementary Table 1. Target information for a large-scale validation of improved genome editing by the U-rich crRNA

Supplementary Table 2. Biased investigation of off-target levels by an on-target probe at potential off-target sites

Supplementary Table 3. Lists of off-target sites for AsCpf1

Supplementary Table 4. Target information for the in vitro and in vivo study

Supplementary Table 5. Probe sequences for Northern blot analysis

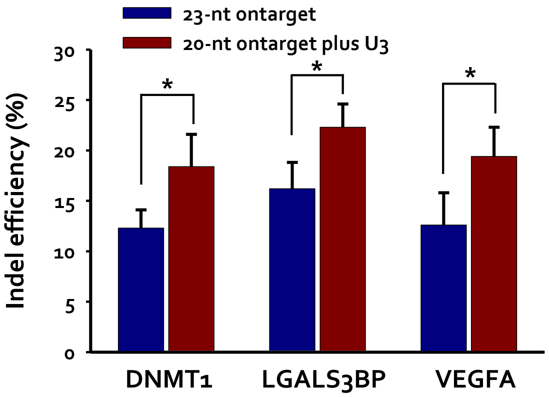

Supplementary Figure 1. Improved AsCpf1 activity by addition of U3 3′-overhang in crRNA. HEK-293T cells were transfected with codon-humanized AsCpf1 vector plus crRNA-encoding PCR amplicons. The configuration of target-recognition part in crRNA includes either 23-nt ontarget sequence or 20-nt ontarget sequence plus UUU (U3). After transfection for 2 days, genomic DNA was prepared and was subjected to PCR amplifications. The amplified target-carrying products were digested with T7E1 enzyme. The indel efficiency was calculated by quantifying digested fragments following resolving the PCR products on 10% SDS-PAGE gels. *; $p<0.05$ (n=3).

*** Target sequence (The underline refers to a PAM sequence).**

DNMT1: 5′-TTTG CTACACACTGGGCATCGGTGGGG-3′

LGALS3BP: 5′-TTTG TGACAGACAGTTCCTGGAGTGCA-3′

VEGFA: 5′-TTTC TCCGCTCTGAGCAAGGCCCACAG-3′

a

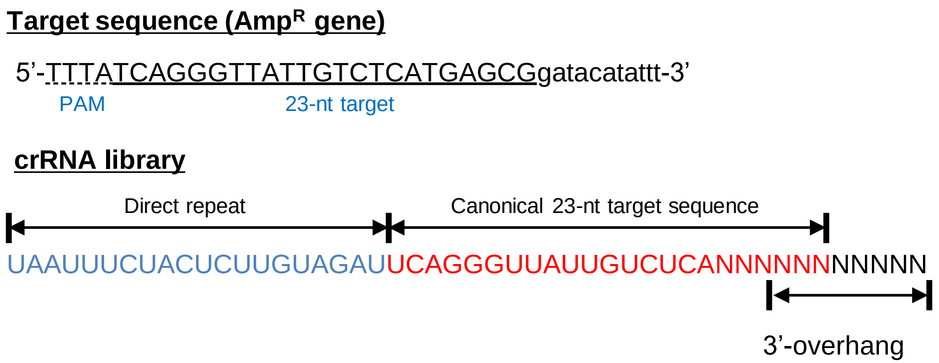

b

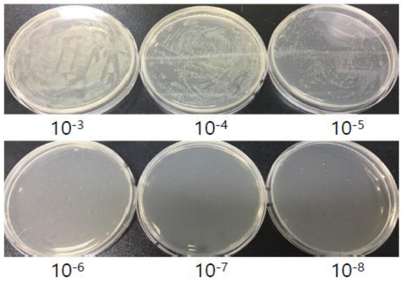

Dilution factor	Number of colony
10^5	1,676
10^6	144
10^7	15
Colony forming unit=1.1558x10^8 CFU/mL	

c

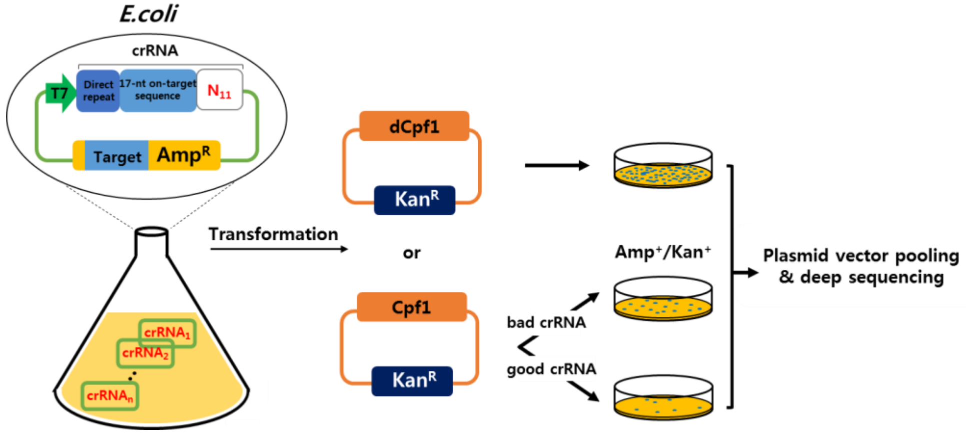

Supplementary Figure 2. Unbiased in vitro analysis to pursue the optimal crRNA configuration. (a) An ampicillin-resistant gene in pET21 plasmid vector was targeted for dsDNA cleavage by AsCpf1 in the presence of crRNA library. crRNA library oligonucleotides with a randomized sequence at positions 18-28 were synthesized and quality-controlled so that each crRNA occupies the equal molar ratio. (b) The oligonucleotide library was cloned into a pET21 plasmid vector using the sequence- and ligation-independent cloning (SLIC) method (Li & Elledge, Methods Mol Biol, 2012). The cloned plasmid vector was used to transform BL21 (DE3) *E. coli* cells and secured a colony forming unit of 1.56x10^8 CFU/mL. (c) The transformed cells were grown to make electro-competent cells carrying the crRNA-encoding plasmid library. The competent cells (2x10^{10} cells/mL) were transformed with either dCpf1 or Cpf1-carrying pET-28a(+) plasmid vector (50-200 ng). The use of dCpf1 vector was to normalize the content of each crRNA in competent cells prior to the transformation using the Cpf1 vector. The transformed cells were plated onto agar plates supplemented with ampicillin and kanamycin plus 0.1 M IPTG. Colonies formed onto each plate were pooled, from which plasmid vectors were purified. The plasmid vectors were subjected to deep sequencing to calculate the frequency of A/T/G/C at each position of crRNA. The basic rationale for our strategy is that a 'good crRNA' cleaves the ampicillin-resistant pET21 vector more efficiently, which, in turn, makes the E.coli cells carrying the 'good crRNA' less prone to survive in the amp(+) plates.

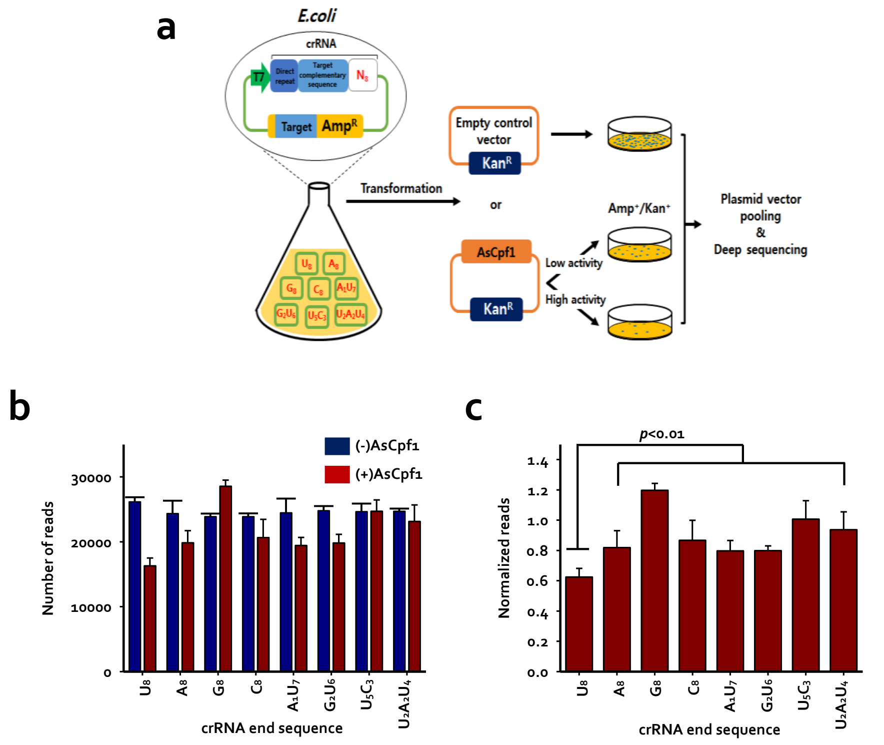

Supplementary Figure 3. Confirmation of the U-rich crRNA as an optimal configuration for AsCpf1 activity. (a) An experimental design to confirm an optimal configuration of crRNA. BL21 (DE3) E. coli cells were transformed with a pET21 vector carrying crRNA with various 8-nt 3′-tails. The crRNA was designed to target a 5′-proximal region of the ampicillin-resistant gene in the plasmid. A colony with a unique crRNA sequence was picked and grown to make electro-competent cells. An equal number of each competent cell was pooled to render crRNA library cells. Pooled competent cells were transformed with pET-28a(+) plasmid vectors with or without AsCpf1 gene. The transformed cells were plated onto agar plates supplemented with ampicillin and kanamycin plus 0.1 mM IPTG. Colonies formed onto each plate were pooled, from which plasmid vectors were purified. The occupancy of each crRNA was measured by deep sequencing analysis. (b) The number of reads is inversely proportional to the efficiency of crRNA. Each read obtained in the absence of AsCpf1 was used to normalize variations of the abundance of crRNA templates inside the competent cells. (c) The normalized read also confirmed that crRNA with the U_8 3′-overhang created the optimal AsCpf1 activity ($p<0.01$, compared with non-U_8 overhang) (n=3).

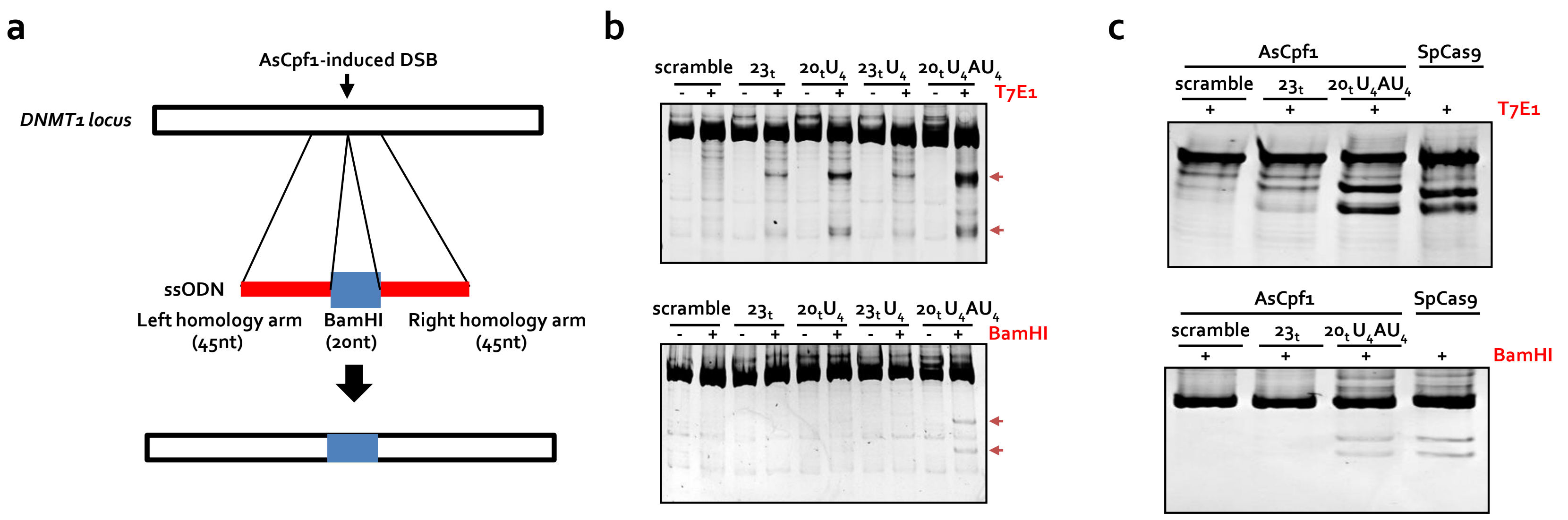

Supplementary Figure 4. Improved knock-in efficiency by crRNA carrying the U-rich 3'-overhang. (a) A DNMT1 locus was targeted for double-strand DNA breakage (DSB) in the presence of crRNA and a donor DNA. The donor DNA was single-strand oligonucleotide (ssODN)-based and designed to carry 45-nt left and right homology arms and a 20-nt mismatch sequence with a BamHI cleavage site in the middle of the homology arms. Knock-in efficiency can be traced by digestion of PCR products carrying the target locus with BamHI. (b) After subjected to indel mutation by CRISPR/Cpf1 system, the target locus was investigated in terms of indel and knock-in efficiency. As expected, crRNA carrying U4AU4 3'-overhang significantly improved indel efficiency at this target site and, more importantly, a traceable level of knock-in efficiency was observed only for the U-rich crRNA, as assessed by the amounts of BamHI-fragments on SDS-PAGE gels (indicated by arrows). (c) The same locus was targeted by both AsCpf1 and SpCas9 with their respective guide RNAs. Although a similar level of indel mutations were observed for AsCpf1 using the U-rich crRNA and SpCas9, knock-in efficiency was higher for SpCas9 than AsCpf1. Nonetheless, it is worthwhile to note that a traceable level of knock-in was confirmed by CRISPR/AsCpf1 system using the U-rich crRNA. All these results were a representative one of three independent experiments.

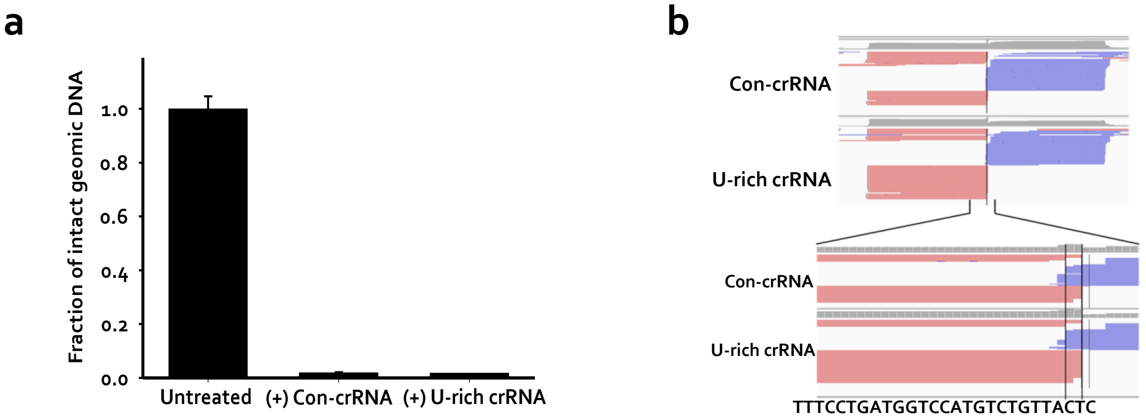

Supplementary Figure 5. Functionality of Digenome-seq off-target analysis. (a) Genomic DNA isolated from HEK-293T cells were subjected to quantitative real-time PCR analysis following digestion with crRNA/AsCpf1 ribonucleoprotein complex. More than 98% of genomic DNA was digested by the complex independently of crRNA types. (b) The cleaved products were then subjected to whole genome sequencing and the sequence data were aligned against human reference genome database (GRCh38.p11). Integrative genomic viewer (IGV) confirmed the typical cleavage pattern at position 18-20 of the non-target strand and 22 of the target strand.

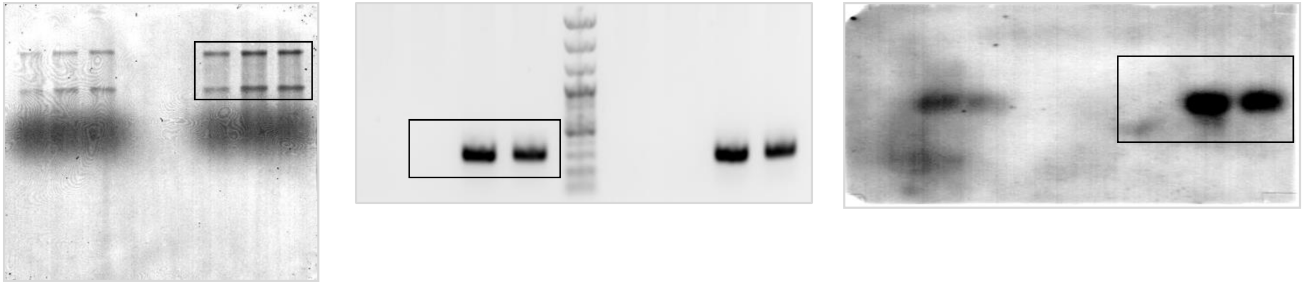

Supplementary Figure 6. Uncropped, original images of blots and gels shown in this study. The images are for Fig. 6a that include the gel images of RNA preparations (left), PCR products (middle), and northern blot (right).

Supplementary Table 1. Target information for a large-scale validation of improved genome editing by the U-rich crRNA[a]

Target No.	Chromosome	Location	Gene name	Target sequence (23nt)	Strand	Type	primer F	primer R	Indel Efficiency (%)		
									Cas9	Cpf1 (Con-crRNA)	Cpf1 (U-rich crRNA)
1	22	16994935	GAB4	CCTGGTGGCTGAGACCAGGGAGG	negative	exon	GTGCTCCCATACCTGTGCTT	CTCACCCAACCTCCTGCTCT	18.3	9.3	7.2
2	21	25603838	MRPL39	ATTTCACAGGACTTTGTTAAAGG	positive	exon	GGCAGGCTGGGAACAGATTAT	GCAGAATCTTGCCTTTCCATTGT	11.2	16.8	20.2
3	14	28794781	LINC01551	ATTTTGAAGTGACCGTACGAGGG	negative	exon	TGCTGTGTACCCCCATTTGA	CTTCACCCAACTTGCACTGG	13.2	1.2	15.6
4	14	28794751	LINC01551	ATAATACACTCTTTACACTGAGG	negative	exon			2.3	42.3	49.6
5	15	24987466	PWAR5	AACAAATCACTGACTAACCAAGG	negative	exon	GTCAGCTACCTTTCCCATGTT	TGAAGTGTTTACGTCCTCCCAT	28.4	3.2	12.8
6	15	24987493	PWAR5	GTGTGGATAAGAATCACCTGAGG	positive	exon			19.2	14.2	25.8
7	3	131069719	NUDT16	GGGGTAGAGGTACTCTACAGGGG	positive	exon	AAAAGATGCTGGACCTTGGC	CAGGATGAGCAGCACTTTGG	34.8	27.1	29
8	3	131069756	NUDT16	GGGGTAGAGGTAGTCTACAGGGG	positive	exon			36.2	32	35.8
9	11	3087968	OSBPL5	GCATTAAGGCCAGCGCTGGGCGG	positive	exon	CGGGGCTCCTCCAAACCTG	CTCCATGGAGGCAGAGAGGC	13.5	3.7	19.5
10	17	3669779	P2RX5-TAX1BP3	CACATAGGCCATTCAGAAACGGG	positive	exon	CTGTAACGCTTAGGCTGCCA	CTGGCCTGTGAAAGGTACAC	28.3	22.1	30.7
11	17	3670244	P2RX5-TAX1BP3	ATTTTAGCAATAACCTTACAGGG	positive	exon			12.2	4.6	17.3
12	20	499271	CSNK2A1	CGTGTTCAAAAACCAAGGCGGGG	positive	exon	TCAAGATGCAGAAAGTGGGC	CCTAGAGCCTGGTGAGACTT	11.2	0	0
13	14	20117733	OR4K17	ACAAGTTCAGAATCACCTTAGGG	negative	exon	ACAGGTCATCCAAGAGCGAG	AGGAGACCCAAGAGCCATGA	12.7	4.6	8.2
14	17	943127	LOC100130876	AAATAACCGTCGGTTTCTTAAGG	positive	exon	CAAGGCTGGGCAGAGTAACTT	TCCCTGGATTTACAGTGGGGTG	12.8	19.2	22.4
15	7	72574897	TYW1B	GATCCGATGCAATTTTGGGAAGG	positive	exon	GTCGTGATATGAGAGGCCCG	TCACCTGGCCCTTGGATTTC	7.5	18.6	24.3
16	13	19073987	LOC107984132	GGAAAGCGCAGAAAAGTAAAAGG	negative	exon	CACTGTCGGAGCTCACATCG	GCCTCCTTCCAGGGTTGATG	13.2	22.1	21
17	19	58005513	LOC100128398	AAGAGTTATTGTCAATAGAAAGG	negative	exon	GCAGAAGCTGGACTTGCCTC	AACCCCCGAGATAGGAAGGG	9.2	0	0
18	19	58005993	LOC100128398	CAAAGAAATGTACTGCCTTACGG	negative	exon			13.2	3.4	15.9
19	7	2434356	CHST12	CCTCTGACTTGACTTCAAACAGG	negative	exon	CCGCACCTGTCTGTTTTTGG	GCTAGAGTGCAATGTCGCGA	0	13.2	15.6
20	16	31193648	FUS	GTGGGTAGGTCCAGTTTGGGGGG	positive	exon	CAACAGTAGGCGGAGAGTGG	GAGGCCAGTTCAAGACCAGC	48.6	15.3	39.5
21	16	31193383	FUS	ACAAAGAAACCAGCAGTGGCAGG	negative	exon			10.2	7.3	11.5
22	7	1233674	UNCX	CCTGAACTCGGGACTCGACCAGG	negative	exon	GCCCTTCAAGCTGTCAGGTA	TCTCGCCACCTGGAACAAAG	3.2	4.3	5.1
23	7	1596749	LOC105375122	CCAACCAGGTACCCTGTGCCAGG	positive	exon	GGGCTCTAATGGCTGTGTGT	CTTTTCCCTCGACCTCCACC	8.2	13	18.6
24	12	908894	WNK1	ACTGGTTATTTCTTGCCAGAGGG	positive	exon	GGGAACTGCCTCCTTGCAGAA	TGGCAAAGTTACATGTCCGC	13.2	2.7	10.9
25	12	909294	WNK1	GAACCCAGTGAAAAATACCAGGG	positive	exon			8.6	1.6	3.6
26	1	25281171	CLIC4	CCCTGGCTACCTCCCCTACCCGG	positive	exon	CGCTTTTCCTAACAGGCTACTCC	GCATTATGCACCAGTTTGGGG	28.6	26.3	19.5
27	1	25281244	CLIC4	GAGGTAGCTTGCCATCTCTCAGG	positive	exon			28.1	19.3	34.8
28	13	19131269	CENPIP1	CTATTCACTTGTGTTACAGGAGG	positive	exon	ACGCCCTAATGAAATTCTAGCCC	GCTGTGCCGGACGATCAAAA	19.4	2.6	3.8
29	13	20002951	ZMYM2	GTAGGCTGCTGTTGGACAGACGG	negative	exon	CCTCTCTGCTATGTTGCTGTTCC	GCCACCTGGACTTGATAGGG	12.6	24.3	20.9
30	5	202864	CCDC127	GGCAAGGGTCTTGATGCATCAGG	positive	exon	AGCACACTGGACATTAGAAACAGG	GATTACAGGCGTGCGCTACC	0	12.2	16.4
31	5	202926	CCDC127	CCGAAAAAATGACTTTTTAGGGG	positive	exon			0	2.3	4.9
32	12	884137	WNK1	ACTCAAGTTGTTCATTCTGCGGG	positive	exon	CCAATTCCTGCGTCTTCCATGCC	CAACATAGCAGAGGCACTGTAG	13.2	7.6	13
33	12	674075	LOC105369597	GCCATGGTGAAGGTGAAATCAGG	positive	exon	GAACCCTTATGGTGGGCTGTGG	GGGATGTCAGTGCTGTTGTGCAG	1.3	2.2	2.5
34	13	18178734	LOC107687186	CTGAATTACAACAAATTGCAAGG	positive	exon	GTCTTTTTCCAGCCTGAGCCAGG	GTCTGCCAAGCTAAGGCTCTCAC	0	10.3	12.2
35	14	20457546	APEX1	AAGAAGGAATGGTAGTTGAGGGG	negative	exon	GCTTCCCCAGTCTTGCCAGTTGT	CCACTGTACCCTTCCTTGTCCGA	28.3	33.5	40.9
36	14	20457653	APEX1	AGCCCAAGATTTTTTATTTGAGG	positive	exon			12.6	25.3	28.3
37	1	25684228	RSRP1	ATATAGGATTTAGAAACCAAGGG	negative	exon	TGTCAGTAGGCCCCCAACTA	GCCTAACTGGCAAATGCCTTA	15.3	12	10.2
38	8	3000119	CSMD1	ACATTTTTAGCTGGCCACTGCGG	negative	exon	TGAACATGGCACCTCTCCTG	TGTTGCGCCTTCAATACTGT	14.3	0	0
39	8	3087237	CSMD1	GAATACCCCCATTCTTCAGGGGG	positive	exon	GTTTGCATGGCCACTAGAAGG	CTCTCACAAAGGCAATGGCAC	11	2.3	18.7
40	9	112718012	INIP	AGAGCAGCGATTGTAAGGAGAGG	negative	exon	ACAGGGCCATCTTGTGACAG	CCGCTAAAGTGCGAATCACG	36.1	3.2	30.4
41	9	14020	DDX11L5	AAAAGATCCCCATGGCCACAGGG	positive	exon	GACGGAGCAGACCCATCTGC	GAGCCTAATGGCCCTTGGCAC	11.3	2.3	14.2
42	3	173963325	NLGN1	AACGAATATTCTCAGACCACAGG	positive	exon	GCCCCCGTATTACCACTCTG	CCAGTGACATGGCCAAGATG	53.2	3.2	6.6
43	1	61097979	LOC105378763	GGGAGGAGAACAGGAAATAAGGG	positive	exon	ACCCCTTCCAATACCATTTGAGA	TGCATAACTCGACAGATACACA	16.8	12.6	27.3
44	1	61097826	LOC105378763	ATTGAAACATATACGTGGTAAGG	negative	exon			6.4	0	0
45	3	173963498	NLGN1	GTCTAATAGAAATATAGTACAGG	negative	exon	GCCCCCGTATTACCACTCTG	CCAGTGACATGGCCAAGATG	12.2	7.3	6.9
46	1	25684090	RSRP1	GCTCTAATGTAAGTATATCCAGG	negative	exon	TGTCAGTAGGCCCCCAACTA	GCCTAACTGGCAAATGCCTTA	7.3	13.2	16.5
47	11	3042164	CARS	CAACAGCCTCACCAGGAACAAGG	negative	exon	GTCCGAGAGACAAGCCAGGG	GATCCTGCTCTCTCTGCCTCC	23.8	11.3	18.3
48	9	14020	DDX11L5	AAAAGATCCCCATGGCCACAGGG	positive	exon	GACGGAGCAGACCCATCTGC	GAGCCTAATGGCCCTTGGCAC	9.3	2.6	6.3
49	12	32393	LOC107987170	GGGTTGCCAGATTAAAAGACAGG	positive	exon	CAGTCAAGTCCAGCAGTTGTCCC	GAGTAGGGTGGCCAGAGGCAG	2.3	3.6	8.6

50	2	32383384	NLRC4	GAGGGAGACACAAGTTGATAGGG	negative	intron	CCCACTCCACTTTGTTCCCAG	TCCTGGGCCCAATCATTCTG	58.4	60.2	69.4
51	20	964362	RSPO4	ACTCATACATCACCTCCTCCAGG	negative	intron	AGGGTTTGAGGGGTTCAGTC	ACTTGACTCCCAACTCAGGC	0	20.3	25.3
52	5	359923	AHRR	CCTTAATAAAGTATAACTTCAGG	negative	intron	TAGGTGGGCAAGAACAGAGG	TTCAGCACAGAGAGGGACAG	16	23.3	25.6
53	19	627446	POLRMT	GAAACTGCCCCAAAACCGGCCGG	negative	intron	CTCCCAGGTTCACTCCATCC	GGCCACGTATTCTAACCAGC	12.5	23.6	29.7
54	19	627491	POLRMT	AGGACTATGTGTGGCCAGTGAGG	negative	intron			13.2	3.2	2.9
55	17	292463	RPH3AL	ATTTTCAAAACAGCCCTATGGGG	positive	intron	TTGGAGAAGCATCACCTGCC	CGGGCTGTGTCCTAACGAAT	11.6	0	0
56	17	292509	RPH3AL	CACAAGGGATCTGAGACTTGAGG	positive	intron			10.6	6.4	11.2
57	4	888480	GAK	ACTCAAGGACTGGCTCAGTGAGG	positive	intron	ACATTCCCAGTGTTCCGTGAG	CATCCAGTCCGTCGCTAAGT	36.4	25.6	28.3
58	4	888530	GAK	CAGAGTCCCGGGAACAAGCCAGG	positive	intron			0	2.3	16.3
59	8	2204833	LOC105377782	TTTACAGCTCTGAGAACTAAACG	negative	intron	CACCCCAACAACTTCTGGGG	AGCATGGTGCAGAATAGTGTGT	3.4	7.6	12.6
60	3	27160152	NEK10	AGACAAGCTGTCTTCCTTCAGGG	negative	intron	GGATTACCTGGGAGGGAGTCA	GGTTGATGTCCACCCCTTCA	19.3	11.4	18.2
61	3	27160372	NEK10	ATCTGAAGATCATTGAAACAGGG	negative	intron			20.3	2.8	18.6
62	20	964345	RSPO4	AAGGAAAGGCTTCCTGGAGGAGG	positive	intron	AGGGTTTGAGGGGTTCAGTC	ACTTGACTCCCAACTCAGGC	20.4	13.6	19.4
63	2	32383454	NLRC4	GTCTCAGTCTTCCTTGTGGGAGG	negative	intron	CCCACTCCACTTTGTTCCCAG	TCCTGGGCCCAATCATTCTG	6.4	4.5	6.1
64	4	42789361	LOC105374431	AGATAAGCGATAGTACATGAGGG	negative	intron	AGTTAATGGGTGCAGCACAC	TCCCAGCAAGTATTCAGCAACA	23.5	3.4	7.6
65	14	19916429	LOC105370393	GCAGTACACCTGAGGGAACAGGG	positive	intron	GCCAGCCCCTGATTCTTCAG	AGTGAATTATGTTGGCTTGGCA	42.1	14.3	19.6
66	14	19916499	LOC105370393	AAGAAAGCTACAGGAAAGCAGGG	positive	intron			5.3	18.3	28
67	22	17678603	BCL2L13	ATTTCCAAGTCAACCTTATGAGG	negative	intron	AGATGACGAGAGCACAGCCT	GGGCCACTAAGTTGCAGGTC	1.3	25.3	29.4
68	22	17678663	BCL2L13	CAAAGTACCTGTTACTTAACAGG	negative	intron			7.3	11.2	34.8
69	12	133140444	ZNF10	AATAAGTCTTACCACGTGTCAGG	positive	intron	GCAGTGGCTCACACCTGTAGTTC	CAGATCTCCAGAATTCTCCTGCTG	0	0	0
70	12	133140502	ZNF10	ATTCCCACAATAACCCTATGAGG	positive	intron			7.6	5.1	16.2
71	12	97515285	RMST	ATAATGCCTTTTAGGTGATAAGG	negative	intron	TAAGAAGCCTATGGGGAGCAG	GGCAAGGTCCCTGAACAGACATG	0	0	9.3
72	12	97515361	RMST	GAGAATAGAAATAAGAAAAAAGG	positive	intron			0	0	6.7
73	3	114911114	LOC101926886	CAAACAAAATAATTGGCTCAGGG	positive	intron	CCTCCCAGCCATGCTTCCTGTTA	AGTTTGGATGCTTGCTCCCTCC	0	0	2.3
74	3	114911188	LOC101926886	CAATCATAGCAGAAGGTGAAGGG	positive	intron			13.6	20.8	28.8
75	4	42789433	LOC105374431	CTTTAAAATGAGGTACTAGGGGG	negative	intron	AGTTAATGGGTGCAGCACAC	TCCCAGCAAGTATTCAGCAACA	35.2	23.4	34.6
76	3	36995716	MLH1	AGGGAATGAAAGTGAAGATGGGG	positive	intron	TGGAGGTTCCAAGGGACCAG	AAGACTCCAGGAGGCCATGG	23.1	19.7	33.6
77	2	23847019	KLHL29	GAGAGACCGCTCAGGCTGGAGGG	negative	intron	AAGCCGAAAGCCTACACCTC	GGACATTCGAAGCCCGTGTA	10.9	2.7	16.7
78	3	36995868	MLH1	GATCAATTTACATCAAACTAGGG	positive	intron	TGGAGGTTCCAAGGGACCAG	AAGACTCCAGGAGGCCATGG	43.2	4.2	9.8
79	4	3343318	RGS12	ATCCCCACAAATACTCTACGAGG	positive	intron	CAGCGTCCCATGCACATTTGGG	GAGAGGACAGCACGGGCAGG	22.5	11.2	10.8
80	3	99413340	COL8A1	GATTCATTCTCAGTGCCATGGGG	positive	intron	GTGGCCAGGGTGGAGGATAAG	CTCTGGCTCCTTTGATACCTCCG	0	9.7	15.4
81	3	99413482	COL8A1	AGGCAATTGCAACCACTGAAGGG	positive	intron			19.2	5.3	16.9
82	5	102556075		GAAATATGACTGGAAGTAAAGGG	negative	intergenic	CCATGACCCACAGAAACTAGAA	TCACCACCATCTCACCTTTG	25.2	20.4	46.1
83	5	102556078		CTTCCAGTCATATTTCTAAAGGG	positive	intergenic			20.6	11.3	17.6
84	5	152068990		CCCTTATTACAATCCTGTGGGGG	positive	intergenic	GGAGGCATTTACAGTGCAGG	AATGCAGGTGAGGCCATTGT	16.2	9.4	15.3
85	5	152068994		CCCCCACAGGATTGTAATAAGGG	negative	intergenic			18.2	21.6	25.4
86	1	88052746		ATCTCCATAACAATCTTTGGGGG	positive	intergenic	GGGGACACATTCAGACCCTA	CTCAGTGTGAACGCGATTGG	19.7	11.1	18.7
87	1	88052777		CTATCCCCATTTTACAGATGAGG	positive	intergenic			11.3	13.6	19.4
88	3	157350012		CTGAGATTTGCGAAGAGTTAGGG	negative	intergenic	GCTCCCTGTTTTGCTCCTTC	CCAACTCCAAGCCAAGCATT	9.4	12	14.3
89	3	157350043		ATTAAATAGAGTCTTTTGAAGGG	negative	intergenic			12.2	8.4	10.9
90	3	128213929		ATATTAATTGCAAGTTTGGGGGG	negative	intergenic	GCTGTGAGGAGAAAAGAGAGCA	GTGGTGAAAGGCCATGAGGG	8.4	2.4	6.2
91	3	128213984		GGCCAAGTGCGAAGTCAGAGGGG	negative	intergenic			19.2	1.2	3.5
92	4	3634902		GGGGTGAACACCCAAGATCCCGG	negative	intergenic	AGGGGACCCCCTGTAGAAC	GGGCCTCAAGTTTGTTTTGC	12.5	16.6	24.7
93	4	3634954		GGGTGGGCTCCTGGCAGGGCAGG	negative	intergenic			37.2	8.6	14.3
94	14	19023974		AAAAGGGGAAAGAGAGAAAGAGG	negative	intergenic	ATGGCTTTTTCAGGATTCCAAACT	GCAGCCCCTACAGAAATGAGT	13.8	16.7	30.1
95	6	254091		AGAAGCATGCAAAACCGGCAAGG	positive	intergenic	GCAGGCTGGTAACTGTGACT	ACCTGCTGCAGAACTGAAGC	28.2	2.3	3.2
96	6	254343		AAGAGGGGAGGTTGACTTTGGGG	positive	intergenic			3.4	3.7	12.3
97	5	97245414		GTCAAATAAAGAAATACACGGGG	positive	intergenic	CCAATGGTGATGAGACAGCGT	GTGGAGGGTGTCCTGGTTCT	21.6	13.2	28.9
98	5	97245470		GTCAAATAAAGAAAAATACGGGG	positive	intergenic			0	0	0
99	20	156154		ATGCATCTCAGTGGTTAACAGGG	positive	intergenic	CTGCCCTCCAGTTGTGACTT	TGCCACAAGGAATCGATGTT	15.3	9.9	13.6
100	8	296459		ACCTCAGGCCTGATCATCAGGGG	negative	intergenic	TGTCTAAGGCCACGACCACAAGC	CCTTCTTGGCACTTCTCGGTGGT	11.2	21.2	23.6
101	4	54520460		CATACAGGGCTCTGTACCCAGGG	negative	intergenic	GGCCCAGAACCTTGCTCTTTGAG	AAGGAGCTGTGCTGTGCAGGTA	23.1	20.4	22.9
102	4	54520536		CAAAGACACTCACCCTGTTGGGG	positive	intergenic			24.1	14.3	26.7

103	5	170399606	AGAACACATACCCCTGGGCCGGG	negative	intergenic	CTGCACCACCACACCTGGCTAAT	AGAACAGAGCAGTGGGCAACAGG	8.4	5.6	14.2
104	5	170399701	ATAATAAAAGTATTTCCTCAGGG	negative	intergenic			11.3	13.2	9.7
105	17	1919439	AGCCGTGGTCAGTGAGAGGCAGG	positive	intergenic	AGAGGGGCACTCGGGAAGAGATA	GGAGGACTTCTTCCCTGTTGGTC	2.3	20.1	26.4
106	17	1919532	GAGCTCATTAGCTTGGGGAGGGG	positive	intergenic			10.4	22.3	21.1
107	4	96592551	GGAAAAGTCATCTGCTACTAGGG	positive	intergenic	TAAACAGGGAAGCGTGGAAGA	TGATGCTTCACCTCAGTGTCT	46.2	15.3	32.6
108	9	7742784	GAAAATAACTAAACTTCCCAGGG	negative	intergenic	ATGATTGGGTTCTGCTGAGGG	AGACCACCTAAAACATTGGCT	34.3	3.5	7.3
109	15	25637364	AATTCTTTAAGTAATTTAAGAGG	negative	intergenic	GGCCTGACCCTCCAGATCTT	GCACTATGCGATCTCCTGGC	12.3	0	0
110	4	96592739	ATTGTATTGTCATAAATTTGGGG	positive	intergenic	TAAACAGGGAAGCGTGGAAGA	TGATGCTTCACCTCAGTGTCT	8.8	6.2	8.4
111	9	7742966	CTTAGTAGTCTCAGAACCAAGGG	positive	intergenic	ATGATTGGGTTCTGCTGAGGG	AGACCACCTAAAACATTGGCT	22.4	14.3	25.3
112	15	25637516	AAAGGAGCACAAGTACAAACAGG	positive	intergenic	GGCCTGACCCTCCAGATCTT	GCACTATGCGATCTCCTGGC	21.5	18.2	25.6
113	18	561716	AATGATGCAGTAATCGTGTAGGG	positive	intergenic	ACAAATCCCCTCATCCCAACG	AAGCTCACTCACCCACCACT	20.6	4.3	19.8
114	5	136515115	ACTTGACATAGTAAGAAACAGGG	positive	intergenic	GCAACAATCGCCATTCCTCACCC	GTGGCCCTCTTATAGCTCTAGG	22.6	23.3	25.9
115	5	136515295	ATAAAAGGAACTATTTACAAGGG	positive	intergenic			18.2	12.2	16.9

[a]Listed information is based on the Genome Reference Consortium Human Build 38 patch release 11 (GRCh38.p11).

Supplementary Table 2. Biased investigation of off-target levels by an on-target probe at potential off-target sites

Target	Reference (Ref) sequence[1)]	Location[2)]	Gene name	crRNA used	# of Total reads	# of Trimmed reads	# of reads with Ref sequence	% of Ref sequence.	# of reads with SNP[3)]	# of reads with indel	% of indel mutations	Sample ID	Primer F	Primer R
				None	113,237	80,713	75,955	94.11	4,758	0	0.00	crRNA_On_N		
Ontarget	[TTTC]TTTCCTGTTTGTCTTGTGTC	63529049	PTK6	Con-crRNA	88,251	77,426	64,657	83.51	4,180	8,589	11.09	crRNA_On_C	CCTCGGGCAGTGATGCTTG	TCCTTACTCCATCGTGTGTGC
				U-rich crRNA	79,625	66,796	50,596	75.75	2,235	13,965	20.91	crRNA_On_U		
				None	37,332	35,248	34,340	97.42	908	0	0.00	crRNA_OF_1_N		
Offtarget_1	[TTTG]TTTtCTGTTTGTCTTGTAGTC	92840367	GRID2	Con-crRNA	34,725	31,542	30,710	97.36	832	0	0.00	crRNA_OF_1_C	CATCCATTCAACCAGTCTCAGT	TCAGTAGAGGAAGAAGGGGAGA
				U-rich crRNA	37,709	36,385	35,391	97.27	994	0	0.00	crRNA_OF_1_U		
				None	48,641	46,258	45,040	97.37	1,218	0	0.00	crRNA_OF_2_N		
Offtarget_2	[TTTG]TTTCCTGTTTGTCTTGT-aC	124222832	CNTNAP5	Con-crRNA	59,834	57,569	56,189	97.60	1,380	0	0.00	crRNA_OF_2_C	AGTTCTAATTGGTCGTTGTG	CTCAACAAACCAGTGACACTTG
				U-rich crRNA	50,501	47,997	46,735	97.37	1,262	0	0.00	crRNA_OF_2_U		
				None	32,085	31,007	30,313	97.76	694	0	0.00	crRNA_OF_3_N		
Offtarget_3	[TTTC]TTTCCTGTTT--CTTtTGTC	19610119	SLC24A3	Con-crRNA	35,845	34,518	33,712	97.66	806	0	0.00	crRNA_OF_3_C	TCCATCACCCCAATTCCAGT	GAAGGGGAGAATGAAGAGAATGA
				U-rich crRNA	36,528	34,980	34,026	97.27	954	0	0.00	crRNA_OF_3_U		
				None	49,137	47,574	46,508	97.76	1,066	0	0.00	crRNA_OF_4_N		
Offtarget_4	[TTTC]TTTCCTGTTTGTCTTGCATcTC	79439750	NRXN3	Con-crRNA	60,548	58,884	57,256	97.24	1,628	0	0.00	crRNA_OF_4_C	TCTCATTCTGTCAAGCCATGT	TGGAAAGCTACCCATGTGATG
				U-rich crRNA	50,080	48,412	46,910	96.90	1,502	0	0.00	crRNA_OF_4_U		
				None	49,908	47,725	46,313	97.04	1,412	0	0.00	crRNA_OF_5_N		
Offtarget_5	[TTTG]TTTCCTGTTTGTtTTGTGTt	31598764	SRD5A2	Con-crRNA	38,098	35,784	34,873	97.45	911	0	0.00	crRNA_OF_5_C	AGGAAGACAGGAAGGAAGGA	AGTGTGTTTCTTGTATGCAGCA
				U-rich crRNA	42,185	40,084	39,026	97.36	1,058	0	0.00	crRNA_OF_5_U		
				None	41,054	40,014	39,225	98.03	789	0	0.00	crRNA_OF_6_N		
Offtarget_6	[TTTA]TTTCtTGTTTGTCTTG-Gta	72315368	[Intergene]	Con-crRNA	33,694	31,815	30,973	97.35	842	0	0.00	crRNA_OF_6_C	CAGAGAAAGGGTAGTGGGACA	AAGGGGAGGGAGAGAGAGAG
				U-rich crRNA	49,518	47,189	45,962	97.40	1,227	0	0.00	crRNA_OF_6_U		
				None	41,120	39,809	38,993	97.95	816	0	0.00	crRNA_OF_7_N		
Offtarget_7	[TTTG]TTTtCTGgTTGTCTTGTTGTC	81529011	GBE1	Con-crRNA	42,874	41,100	39,805	96.85	1,295	0	0.00	crRNA_OF_7_C	GATTGGAGAGTTCAGTCCAT	GCAAGCCTCATGAGAACCACA
				U-rich crRNA	58,045	55,784	54,500	97.70	1,284	0	0.00	crRNA_OF_7_U		
				None	43,325	42,845	41,787	97.53	1,058	0	0.00	crRNA_OF_8_N		
Offtarget_8	[TTTG]TTT--TGTTTGTCTTGTtTt	21505926	[Intergene]	Con-crRNA	36,415	34,550	33,556	97.12	994	0	0.00	crRNA_OF_8_C	AAGGTGGGAGGATTGCTTGA	TACATGGTGGTTTTGCAGGC
				U-rich crRNA	52,409	51,177	49,976	97.65	1,201	0	0.00	crRNA_OF_8_U		
				None	48,023	46,698	45,793	98.06	905	0	0.00	crRNA_OF_9_N		
Offtarget_9	[TTTG]TTTCCTGTTTcTCT--TtTC	141212565	TMEM178B	Con-crRNA	36,605	34,401	33,333	96.90	1,068	0	0.00	crRNA_OF_9_C	AGTTGTCACCAGGCCCAATA	AAGTGAGGTTCCTGGGGATG
				U-rich crRNA	42,229	41,007	39,671	96.74	1,336	0	0.00	crRNA_OF_9_U		

1) Four letters in parenthesis indicate a PAM sequence. Lower cases and dashes indicates mismatch sequences and bulges, respectively.
2) Location is based on the Genome Reference Consortium Human Build 38 patch release 11 (GRCh38.p11).
3) The occurrence of SNP was monitored in the investigated alleles by comparing with the sequences of non-treated alleles. These single-nucleotide variations identically observed between Cpf1-treated and non-treated alleles were deemed to be SNP. Those SNPs were taken into account and excluded when calculating off-target frequencies.

Supplementary Table 3. Lists of off-target sites for AsCpf1

Con-crRNA				U-rich crRNA			
Chromosome	Location	DNA Cleavage score	Target sequence	Chromosome	Location	DNA Cleavage score	Target sequence
Chr19	43767815	15.8	TTTACTGATGGTCCAaacaTcTaA	Chr19	43767815	11.3	TTTACTGATGGTCCAaacaTcTaA
Chr1	10179491	10.4	TTTACTGATGGTCCATccCTtTTA	Chr6	141623485	10.2	TTTGCTGATGGTCtATagCTaTcA
Chr19	43263943	9.1	TTTACTGATGGTCCAaacaTcTaA	Chr6	138526960	8.7	TTTCCTGATGGTCtgTtTtTGTag
Chr1	177026436	8.4	TTTGCTGATGGTCgATtTaTacTg	Chr19	10244444	7.8	TTTCCTGATGGTCCATGTCTGTTA
Chr19	10244444	8.2	TTTCCTGATGGTCCATGTCTGTTA	Chr19	43263943	7.7	TTTACTGATGGTCCAaacaTcTaA
Chr6	16517291	7.7	ATTCCTGATGaTCCATGcCTGcat	Chr5	163936302	7.3	TTTCCTGATGGTCtATtTtTccTt
Chr19	43416520	6.9	TTTACTGATGGTCCAaacaTcTaA	ChrX	92673750	7.3	TTTCCTGATGGTCCAcagaTacTA
Chr5	39969437	6.7	TCTCCTGATGGTCCATacCTGTTA	Chr21	44021964	6.9	TTTCCTGATGGTCtAcacCTGTTg
Chr2	233034313	6.2	TTTAgTGATaGTCCATGTCTGcag	Chr19	43435385	6.8	TTTACTGATGGTCCAaacaTcTaA
Chr19	43353967	6	TTTACTGATGGTCCAaacaTcTgA	Chr1	10179491	6.7	TTTACTGATGGTCCATccCTtTTA
Chr6	141623485	5.7	TTTGCTGATGGTCtATagCTaTcA	Chr19	43377706	6.6	TTTACTGATGGTCCAaacaTcTaA
Chr13	70187460	5.6	TTTCCTGATGGTCCAcactTGTTg	Chr3	122020326	6.5	TTTACTGATGaTCtATaTtTacTA
Chr21	44021964	5.6	TTTCCTGATGGTCtAcacCTGTTg	Chr19	43416520	6.4	TTTACTGATGGTCCAaacaTcTaA
Chr5	163936302	5.6	TTTCCTGATGGTCtATtTtTccTt	Chr1	177026436	6.3	TTTGCTGATGGTCgATtTaTacTg
Chr19	43377706	5.5	TTTACTGATGGTCCAaacaTcTaA	Chr1	186592956	5.8	TTTCCTcATGGTCCATGTCaGgac
ChrX	81346070	5.4	TTTCCTGATGGTCCAcacCTaTTg	Chr16	75745894	5.7	TTTTCTGATGGTCCATacCTGTTA
ChrX	115862098	5.1	TTTCaTGATGGTCCATacCTGTTA	Chr6	16517291	5.7	ATTCCTGATGaTCCATGcCTGcat
Chr1	213377379	5.1	TTTCCTGATGGTCCATGTCTGaat	Chr19	43353967	5.6	TTTACTGATGGTCCAaacaTcTgA
Chr4	151678397	4.7	TTTGCTGATGGTCtcTtTaacTTA	ChrX	115862098	5.6	TTTCaTGATGGTCCATacCTGTTA
Chr1	89819958	4.5	TTTCCTGATGGcCCATacCTGTTA	Chr1	236623991	4.9	TTTACTGATGaTCCATGTCTaaac
Chr1	242619943	4.3	TTTGgTGATGGTCtATaTCaGagA	Chr13	70187460	4.9	TTTCCTGATGGTCCAcactTGTTg
Chr2	89591302	4.2	TTTCCTGATGGTCCAcacCTtTTg	Chr1	213377380	4.9	TTTCCTGATGGTCCATGTCTGaat
Chr13	81006434	4	TTTCCTGATGGTCCAcactTGTgg	Chr1	238343056	4.7	TTTCCTGATGGTCCAcacCTaTTg
ChrX	97546178	3.9	TTTCCTGATGGTCCAcGcCTGTTA	Chr5	35891131	4.5	TTTCCTGATGGTCtAcacCTGTTg
Chr22	27745385	3.8	TTTCCTGATGGTCCAcactTaTTA	ChrX	97546178	4.2	TTTCCTGATGGTCCAcGcCTGTTA
Chr3	96050499	3.8	TTTCCTGATGGTCCATactTGTTg	Chr17	53836590	4.1	TTTACTGATGGTCCATacCTcgTA
Chr1	238343056	3.4	TTTCCTGATGGTCCAcacCTaTTg	ChrX	94580341	4.1	TTTCCTGATGGTCCAcactTGTTg
Chr3	195961223	3.4	TTACCTGATGtTCCATGTCcagTg	Chr2	89591301	4	TTTCCTGATGGTCCAcacCTtTTg
Chr13	82088076	3.4	TTTCCcGATGGTCCAcaTCTGTTA	Chr12	58560889	3.8	TTTCCTGATGGTCtAcacCTGTTg
Chr17	53836590	3.2	TTTACTGATGGTCCATacCTcgTA	Chr13	81006434	3.8	TTTCCTGATGGTCCAcactTGTgg
Chr1	146123498	3.2	TTTCCTGATGGTCCAcacCTGTTg	Chr6	154888710	3.7	TTTACTaATGGTCCAaaTCctTcA
Chr2	4463241	3.2	TTTAgTGATGGTCCcTaTtTcTTc	Chr4	151678397	3.6	TTTGCTGATGGTCtcTtTaacTTA
Chr3	142979810	3.1	TCTCCTGATGGTCCAcGcCTGTTA	Chr7	112920853	3.4	TTTGCTGATGGTCtgTaTCTGTgA
Chr4	125429316	3	TTTCCTGATGGTCCAcacCTaTTg	Chr8	34932811	3.3	TCTACTGATGGTCCtTaTtTGTTg
Chr7	68777908	3	TTTCCTGcTGGTCCATGTCTaaTA	Chr4	31284788	3.2	TTTCCTGATGaTCtATcTaTagTA
Chr1	236623993	3	TTTACTGATGaTCCATGTCTaaac	Chr3	135976149	3.1	TTTGCTGATGGTCCcctTCTcccA
ChrX	92676365	3	TTTCCTGATGGTCCATacCTGTTA	ChrX	130910822	3.1	TCTCCTGATGaTCCAcaTCTGTTA
Chr11	26124230	2.9	TTTCCTGATGGTCCAcaTCTGTTA	Chr3	96050498	3	TTTCCTGATGGTCCATactTGTTg
Chr4	84421821	2.8	TTTCCTGATGGTCCAcacCTtTTg	Chr7	68777909	2.9	TTTCCTGcTGGTCCATGTCTaaTA
Chr6	138526961	2.6	TTTCCTGATGGTCtgTtTtTGTag	Chr4	84421821	2.9	TTTCCTGATGGTCCAcacCTtTTg
Chr5	35891132	2.6	TTTCCTGATGGTCtAcacCTGTTg	ChrX	92676365	2.8	TTTCCTGATGGTCCATacCTGTTA
				Chr5	178329	2.7	TTTCCTGATGGTCCAcacCTGcTg
				Chr11	26124230	2.7	TTTCCTGATGGTCCAcaTCTGTTA
				Chr6	147610255	2.7	TTTCCTGATGGTCCAcacCTGcTg
				Chr1	89819958	2.6	TTTCCTGATGGcCCATacCTGTTA
				Chr9	35488299	2.5	TTTCCTGATGGTCCAcacaTGTTA

Supplementary Table 4. Target information for the *in vitro* and *in vivo* study

Figure	Gene name	Chromosome	Target sequence[1)]	Location	Strand	Type	Primer F	Primer R	Description
1a, 2b, 2c, 2g, 4b	DNMT1	19	**[TTTC]**CTGATGGTCCATGTCTGTTACTC	1013370	negative	intron	CTGGGACTCAGGCGGGTCAC	CCTCACACAACAGCTTCATGTCAGC	
1b, 1c, 1e, 2d, 2e, 6c, Supplementary Fig. 1	DNMT1	19	**[TTTG]**CTACACACTGGGCATCGGTGGGGG	10207808	negative	intron	AAGCAAATCCACCTGCCTCG	CCTCCCCTAGCCCTTTCAGG	
2f	VEGFA	6	**[TTTC]**TCCGCTCTGAGCAAGGCCCACAG	43781959	negative	exon	CTAGCCAGTGCTGCCTCTTT	CGCTCGCTCACTCTCTTTCT	
Supplementary Fig. 1	TP53	17	**[TTTC]**GACATAGTGTGGTGGTGCCCTAT	7674841	positive	exon	CAGATAGCGATGGTGAGCAG	GGGAGGTCAAATAAGCAGCAGG	
Supplementary Fig. 1	LGALS3BP	17	**[TTTG]**TGACAGACAGTTCCTGGAGTGCA	78972059	positive	exon	ACTGAAGGCCGTGGACACCT	CTTGTCCTGGAAGAGGAAGC	
2f, 5a, 6b	INIP	9	**[TTTA]**AGAGCAGCGATTGTAAGGAGAGG	112718012	negative	exon	ACAGGGCCATCTTGTGACAG	CCGCTAAAGTGCGAATCACG	Target1 (Fig.5a)
2f, 5c	LOC105370393	14	**[TTTA]**AAGAAAGCTACAGGAAAGCAGGG	19916499	positive	intron	GCCAGCCCCTGATTCTTCAG	AGTGAATTATGTTGGCTTGGCA	Target1 (Fig.5c)
5a	KLHL29	2	**[TTTA]**GAGAGACCGCTCAGGCTGGAGGG	23847019	negative	intron	AAGCCGAAAGCCTACACCTC	GGACATTCGAAGCCCGTGTA	Target2 (Fig.5a)
5a	KLHL29	2	**[TTTA]**GGGAGACAGGGAGAAGTGAGAGG	23847166	negative	intron			Target3 (Fig.5a)
5b	KIF26B	1	**[TTTA]**CCCCTGCATTGCCATGAGCCCCC<br>**[TTCC]**GGGGGCTCATGGCAATGCAGGGG	245687161	positive<br>negative	exon	CTTTCAACAAAGCAGCCCCC	TGCTCTGGTCTCAGCATTCG	Target1
5b	CAV1	7	**[TTTA]**CCCGAGTCCTGGGGACAGTCCCC<br>**[TCCC]**GGGGACTGTCCCCAGGACTCGGG	116525483	positive<br>negative	intron	TGAGATTGGGTCTGTTGGGC	TGAGATTGGGTCTGTTGGGC	Target2
5b	ITGB5	3	**[TTCC]**CCGCAGTGACACTCGCCATGGCC<br>**[TTTA]**GGCCATGGCGAGTGTCACTGCGG	124773887	positive<br>negative	exon	TTGGTAAGAATGCGGCTCCC	CATAACCATCTGGTGCCCCA	Target3
5c	COL8A1	3	**[TTTA]**GATTCATTCTCAGTGCCATGGGG	99413340	positive	intron	GTGGCCAGGGTGGAGGATAAG	CTCTGGCTCCTTTGATACCTCCG	Target2
5c	COL8A1	3	**[TTTA]**AGGCAATTGCAACCACTGAAGGG	99413482	positive	intron			Target3

1) Four letters in parenthesis indicate a PAM sequence.

Supplementary Table 5. Probe sequences for Northern blot analysis

Target	Sequence
DNMT1 target 3 on-target	5′-AATTTCTACTCTTGTAGATCTGATGGTCCATGTCTGTTACTC-3′
DNMT1 target 3 U-rich	5′-AATTTCTACTCTTGTAGATCTGATGGTCCATGTCTGTTATTTTATTTTTT-3′
